# The Effect of Zinc Gluconate Supplementation on the Intestinal Microbiota and Growth Performance of Yorkshire Pigs

**DOI:** 10.3390/ani16111607

**Published:** 2026-05-25

**Authors:** Yan Zhu, Jiawei Lu, Jingwen Fan, Linyuan Shen, Li Zhu, Ya Tan, Yihui Liu, Mailin Gan

**Affiliations:** 1 State Key Laboratory of Swine and Poultry Breeding Industry, College of Animal Science and Technology, Sichuan Agricultural University, Chengdu 611130, China; 2024302105@stu.sicau.edu.cn (Y.Z.); 2024302131@stu.sicau.edu.cn (J.L.); 202400529@stu.sicau.edu.cn (J.F.); shenlinyuan@sicau.edu.cn (L.S.); zhuli@sicau.edu.cn (L.Z.); 2 Key Laboratory of Livestock and Poultry Multi-Omics, Ministry of Agriculture and Rural Affairs, College of Animal Science and Technology, Sichuan Agricultural University, Chengdu 611130, China; 3 Farm Animal Germplasm Resources and Biotech Breeding Key Laboratory of Sichuan Province, Sichuan Agricultural University, Chengdu 611130, China; 4 Institute of Animal Husbandry and Veterinary, Guizhou Academy of Agricultural Science, Guiyang 550005, China; tanya_lee@126.com; 5 Sichuan Province General Station of Animal Husbandry, Chengdu 610066, China

**Keywords:** 16S rRNA gene, zinc gluconate, Yorkshire Pigs, gut microbiota, growth performance, energy metabolism, *Clostridia_UCG-014*

## Abstract

The gut microbiota significantly influences the healthy growth and development of animals. However, the alterations in the gut microbiota of Yorkshire Pigs mediated by specific trace elements, such as zinc gluconate, and their associations with growth phenotypes remain to be elucidated. This study aimed to investigate the effects of dietary zinc gluconate supplementation on the gut microecology of Yorkshire Pigs. Twenty Yorkshire Pigs were randomly divided into a control group (NC) and a treatment group (GZn, fed a diet containing 0.1% zinc gluconate) for 52 days. The diversity, composition, and potential functions of fecal microbiota were evaluated via 16S rRNA gene sequencing. The results showed that dietary zinc gluconate supplementation altered the gut microbiota of Yorkshire pigs, evidenced by distinct community clustering in beta diversity and increased alpha diversity (species richness and diversity). In terms of the composition of taxonomic units, a significant beneficial succession occurred among the core bacterial genera: potential probiotic genera (such as *Clostridia_UCG-014*) that were significantly enriched, while genera associated with negative correlations with growth traits or potentially pathogenic (such as *Streptococcus* and *Escherichia-Shigella*) were effectively suppressed. Although Firmicutes and Bacteroidota remained the shared dominant phyla, the abundance of core genera underwent a significant succession. Spearman correlation analysis revealed that potential probiotic genera positively correlated with body weight and body length (such as *Clostridia_UCG-014*, *Methanobrevibacter*, and *Oscillospira*) were significantly enriched in the GZn group; conversely, genera negatively correlated with growth traits (such as *Streptococcus* and *Escherichia-Shigella*) were enriched in the NC group. Furthermore, KEGG pathway functional prediction indicated that the gut microbiota of the GZn group was predicted to be enriched in fundamental metabolic functions such as amino acid metabolism and energy metabolism. In conclusion, dietary zinc gluconate supplementation can effectively improve the growth phenotypes of Yorkshire Pigs, and this improvement is associated with shifts in the gut microecological structure and predicted microbial metabolic functions.

## Simple Summary

The gut microbiota comprises a vast community of bacteria essential for animal health and development. However, the precise mechanisms by which specific micronutrients, such as zinc gluconate, promote growth by modulating this microbial community remain poorly understood. This study aimed to investigate whether dietary zinc gluconate supplementation could enhance growth performance by optimizing the gut microenvironment in pigs. Our findings revealed significant increases in body weight and body length in the treated group. These phenotypic improvements were associated with an enrichment of beneficial bacteria that facilitate nutrient absorption and energy harvest, alongside a significant reduction in potential pathogens. We conclude that zinc gluconate supplementation is associated with changes in gut microbiota composition, thereby promoting growth. This research provides a safe and sustainable nutritional intervention for the livestock industry, potentially reducing the reliance on antibiotics. Such strategies not only improve agricultural economic outcomes but also contribute to the enhancement of global food safety.

## 1. Introduction

The gut microbiota, comprising a vast array of bacterial species [1], forms a complex community and significantly modulates intestinal health in humans and monogastric animals [2]. GM regulates essential physiological processes, such as energy metabolism, nutrient digestion and absorption, and immune modulation [3,4]. In the livestock sector, the study of the gastrointestinal microbiota shows great potential for improving the nutritional and immune activities of the host and improving livestock productivity [5].

Zinc is one of the important trace elements in the human body. It participates in the metabolism of various enzymes in the body and is essential for many enzymes and macromolecules. Zinc also participates in the metabolism of various substances in the body, such as the metabolism of proteins and amino acids. It is also related to the body’s immunity, growth and development, etc. It also has the functions of regulating appetite, stabilizing biofilm, anti-ulcer and treating colds [6,7]. In modern intensive pig farming, high doses of inorganic zinc, particularly zinc oxide (ZnO), have been widely utilized to prevent post-weaning diarrhea and promote growth [8]. However, the long-term and excessive application of inorganic zinc has raised significant concerns, including severe environmental pollution through fecal excretion and the potential co-selection of antibiotic-resistant bacteria in the gut [9]. Furthermore, pharmacological levels of ZnO may lead to intestinal mucosal damage and disrupt the mineral balance in the host [10]. Consequently, there is an urgent need to identify ‘gentle and efficient’ organic zinc sources, such as zinc gluconate, which offer higher bioavailability and lower environmental impact at reduced inclusion levels. Zinc gluconate is an organic zinc supplement, which has little irritation to the gastric mucosa, is easily absorbed in the body, and has a high absorption rate and good solubility [11]. Zinc and zinc homeostasis [12] are also important for the structure and function of the intestinal mucosal barrier [13,14].

Body length and weight are important indicators for evaluating animal growth and development in the field of animal husbandry [15,16], and the weight gain can show the changes in animal weight during the experiment. A study in pigs found that early attention and alterations to the gut microbiota can affect the animal’s phenotype [17]. In the past ten years, the rise of 16S sequencing technology has enabled us to better study the impact of GM on the body [18,19], and we can use 16S sequencing technology to analyze the correlation between Yorkshire Pigs phenotype and intestinal microbial community [20]. Therefore, 16S has become the most mature and advanced technology for GM research. Yorkshire pigs are spread all over the world because of their strong fecundity, thin backfat, lean meat and good meat quality [21]. This study aimed to investigate whether dietary zinc gluconate supplementation could comprehensively improve the growth performance of Yorkshire pigs, and to elucidate the impact of this dietary intervention on the gut microbiota. In this study, we used a porcine model to investigate the microbiota response to dietary zinc gluconate supplementation. Our research will contribute to the understanding of the mechanisms behind zinc gluconate’s regulation of gut health.

## 2. Materials and Methods

### 2.1. Animals and Sample Collection

The animals used in this experiment were obtained from a commercial pig breeding company located in Sichuan Province, China. All boars were housed under standard, similar environmental conditions with an ambient temperature maintained at approximately 17–22 °C, and were provided ad libitum access to feed and water. The dietary contents of crude protein, trace elements, vitamins, and energy met or exceeded the nutrient requirements recommended by the National Research Council (NRC, 2012) for the respective production stages. A total of 20 Yorkshire boars with an initial average age of 127.9 ± 9.35 days were selected. To ensure statistical validity, the pigs were allocated into two dietary treatments (n = 10 per group) based on a randomized complete block design, with initial body weight serving as the blocking factor. Each pig was individually housed in a standard pen, which served as the experimental unit. The two groups included: a control group (NC) fed a basal diet without zinc gluconate supplementation, and a treatment group (GZn) fed the basal diet supplemented with 0.1% zinc gluconate, with an estimated daily feed intake of approximately 2.5 kg per boar. All boars enrolled in this study were healthy, free from clinical signs of diseases such as diarrhea, and had no history of antibiotic treatment prior to the experiment. The experimental diets were provided continuously for 52 days until the Yorkshire Pigs were slaughtered at 180 days of age. Fresh fecal samples were collected randomly from individual pigs immediately upon defecation (within one minute) to ensure sample freshness. The collected samples were promptly placed into 2 mL sterile centrifuge tubes and transported on ice. Upon arrival at the laboratory, all samples were stored at −80 °C prior to subsequent DNA extraction.

### 2.2. Microbiota Analysis Based on 16S rRNA High-Throughput Sequencing

This study employed high-throughput sequencing of the microbial 16S rRNA gene to determine the diversity and composition of the microbiota in the fecal samples of 10 Yorkshire Pigs in each group (n = 10 per group).

### 2.3. Statistical Analysis

The method of sequencing data analysis was the same as previously reported. Briefly, paired-end sequences were merged using FLASH v.1.2.7 (http://ccb.jhu.edu/software/FLASH/ (accessed on 20 July 2025)). Raw sequences were quality controlled and data cleaned using QIIME v.1.7.0 (http://qiime.org/index.html (accessed on 20 July 2025)). Tags were compared with a reference database (Gold database, http://drive5.com/uchime/uchime_download.html (accessed on 25 July 2025)) using the UCHIME algorithm. Sequences with ≥97% similarity were classified into the same operational taxonomic unit (OTU) using Uparse software v.7.0.1001 (http://drive5.com/uparse/ (accessed on 25 July 2025)). Representative sequences for each OTU were selected and each representative sequence was taxonomically annotated using the RDP classifier (http://sourceforge.net/projects/rdp-classifier/ (accessed on 30 July 2025)). The number of sample sequences was used for normalization to obtain the normalized OTUs with the least number of sequences. Subsequent analyses were based on normalized OTUs.

Alpha diversity indicators were calculated using Qiime2 (qiime2-2020.6), which includes coverage indices for observed species, Chao1, Shannon, Simpson, and Good; observed species index measured the number of species per sample; Chao1 index estimates species Richness; Good’s coverage index measures sequencing depth; Shannon and Simpson indicate diversity and evenness of species distribution. Qiime2 was used to calculate beta diversity based on unweighted UniFrac distances using principal coordinates analysis (PCoA). PCoA analysis was performed using R software (Version 3.5.3), ggplot2 package, extrafont package, grid package and ade4 package in Qiime2. The phylogeny among the identified OTUs was calculated by an unweighted pairwise method using arithmetic mean (UPGMA) clustering. Linear discriminant analysis (LDA) effect size (LEfSe) and T-test statistical analysis were used to identify significant differences in abundance between different sample groups [22]. LEfSe analysis was performed using an LDA score of 3 [23]. OTU functions were annotated against the Greengenes database using PICRUST2 (v2.3.0_b).

## 3. Results

### 3.1. Comparison of Phenotypic Indicators

Based on preliminary data, we selected the body size data of Yorkshire Pigs weighing between 90–120 kg for analysis, including 10 in the NC group and 10 in the GZn group. Before feeding, there was no significant difference in body weight between the NC and GZn groups (70 ± 3.44 kg for GZn vs. 70 ± 3.44 kg for NC). After 52 days of feeding, the pre-slaughter live weight was 113.22 ± 5.47 kg vs. 101.83 ± 8.55 kg (GZn vs. NC), torso weight was 83.94 ± 3.98 kg vs. 76.92 ± 6.64 kg, weight gain was 41.61 ± 5.08 kg vs. 31.83 ± 9.33 kg, head weight was 6.47 ± 0.29 kg vs. 5.99 ± 0.36 kg, and body length was 118.89 ± 4.25 cm vs. 114.33 ± 3.73 cm (Table 1). The pre-slaughter live weight, torso weight, weight gain, and head weight in the GZn group were significantly higher than those in the NC group (*p* < 0.05). Compared with the NC group, the body length in the GZn group showed an increasing trend, although this difference was not statistically significant (*p* = 0.0685). Furthermore, no significant differences were observed in the visceral organ weights between the NC and GZn groups, suggesting that zinc gluconate at this dosage had no obvious adverse effects on visceral development in Yorkshire Pigs, demonstrating good safety and tolerability. Notably, dietary supplementation with zinc gluconate not only had a significant effect on pig weight gain but also showed a significant effect on increasing body length.

### 3.2. The Effect of Zinc Gluconate on the Diversity of Yorkshire Pig Gut Microbiota

**Figure 1:**
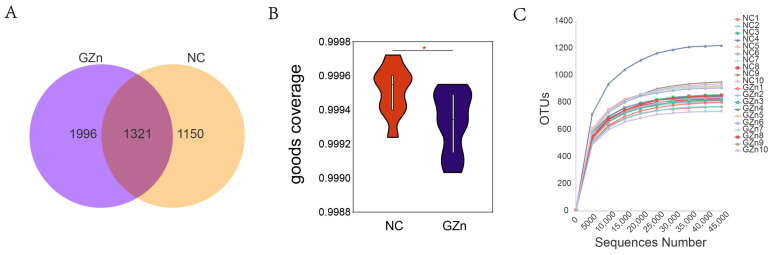
DNA sequence data analysis. (**A**) Venn diagram. Numbers in the graph show unique or shared OTUs for each group. (**B**) shows the sequencing depth of NC group and GZn group. (**C**) Rarefaction analysis of different stool samples at 97% sequence identity. * indicates a statistically significant difference (*p* < 0.05).

In this study, the 16S rRNA genes of fecal samples from a total of 20 Yorkshire Pigs in two groups were sequenced. After taxonomic assignment, a total of 4467 OTUs were obtained based on 97% nucleotide sequence similarity, with 2471 and 3317 OTUs obtained in the NC and GZn groups, respectively. The number of shared OTUs between the NC and GZn groups was 1321, while the number of OTUs unique to the NC and GZn groups were 1150 and 1996, respectively, showing a clear increasing trend in the latter (Figure 1A). The Good’s coverage index values for all samples in both the NC and GZn groups exceeded 99% (Figure 1B). The rarefaction curves tended to plateau when the number of valid sequences exceeded 45,000 (Figure 1C). The sequencing results indicated that both sequencing depth and sequence quantity met the requirements for analysis.

### 3.3. Analysis of Microbial Diversity Index

**Figure 2:**
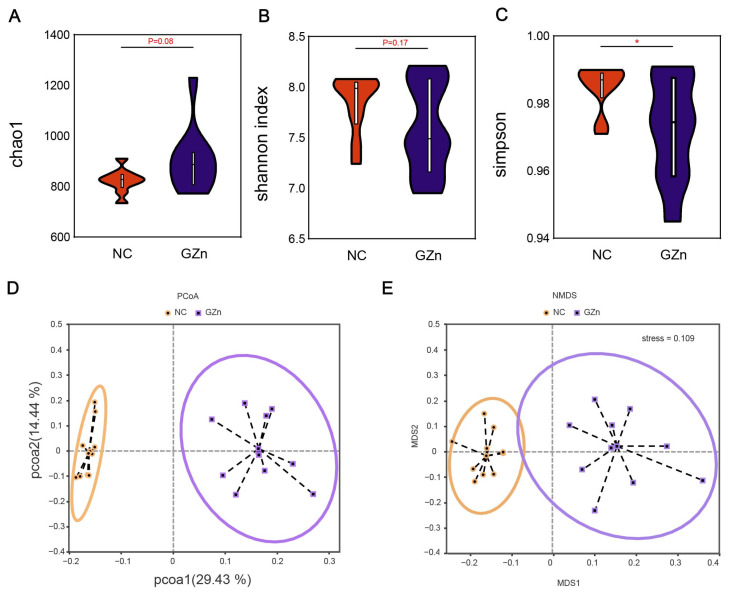
Differences in gut microbiota between NC group and GZn group. Bacterial alpha diversity is based on Shannon diversity, Simpson diversity and Chao1 index. * indicates a statistically significant difference (*p* < 0.05). (**A**) Chao1 index represents species richness. (**B**) Shannon diversity represents microbial community diversity and is positively correlated with it. (**C**) Simpson diversity represents microbial community diversity and is negatively correlated with it. (**D**) Differences in principal coordinate analysis (PCoA) between NC and GZn groups of GM structures. The yellow dots represent the samples of the NC group, and the purple square dots represent the samples of the GZn group. The distance between two points represents the difference in GM. (**E**) Differences in the non-metric multidimensional alignment (NMDS) of the NC group and the GZn group of GM structures.

To evaluate Alpha diversity, species richness and community diversity were assessed using the Chao1, Shannon, and Simpson indices (Table 2). The Shannon index was 7.84 ± 0.28 for the NC group and 7.59 ± 0.43 for the GZn group. The Simpson index was 0.985 ± 0.007 for the NC group and 0.972 ± 0.015 for the GZn group. The Chao1 values ranged from 734.48~910.43 for the NC group and 772.11~1230.49 for the GZn group. The results demonstrated that the Simpson index in the GZn group was significantly lower than that in the NC group (*p* = 0.0309) (Figure 2C), indicating higher species diversity in the gut microbiota of the GZn group. In addition, the Chao1 index in the GZn group showed an increasing trend compared to the NC group (*p* = 0.0817, Figure 2A), suggesting that zinc gluconate intervention helps enhance the species richness of the gut microecology. The Shannon index in the GZn group was slightly lower than that in the NC group, but the difference was not statistically significant (Figure 2B). Based on the sample OTU species and their relative abundances, a PCoA plot was generated using the Bray–Curtis algorithm. The results showed that the GZn group mainly clustered on the right side of the coordinate axis, while the NC group mainly clustered on the left. The samples from the NC and GZn groups exhibited relatively clear separation, with the first two principal coordinates explaining 29.43% and 14.44% of the total variance, respectively (Figure 2D). This indicates differences in the gut microbiota structure between the NC and GZn groups. Furthermore, the Non-metric Multidimensional Scaling (NMDS) plot based on the Bray–Curtis algorithm also confirmed structural differences in the gut microbiota between the NC and GZn groups (Figure 2E).

### 3.4. Analysis of Community Composition Between Zinc Gluconate Treatment Group and Control Group

**Figure 3:**
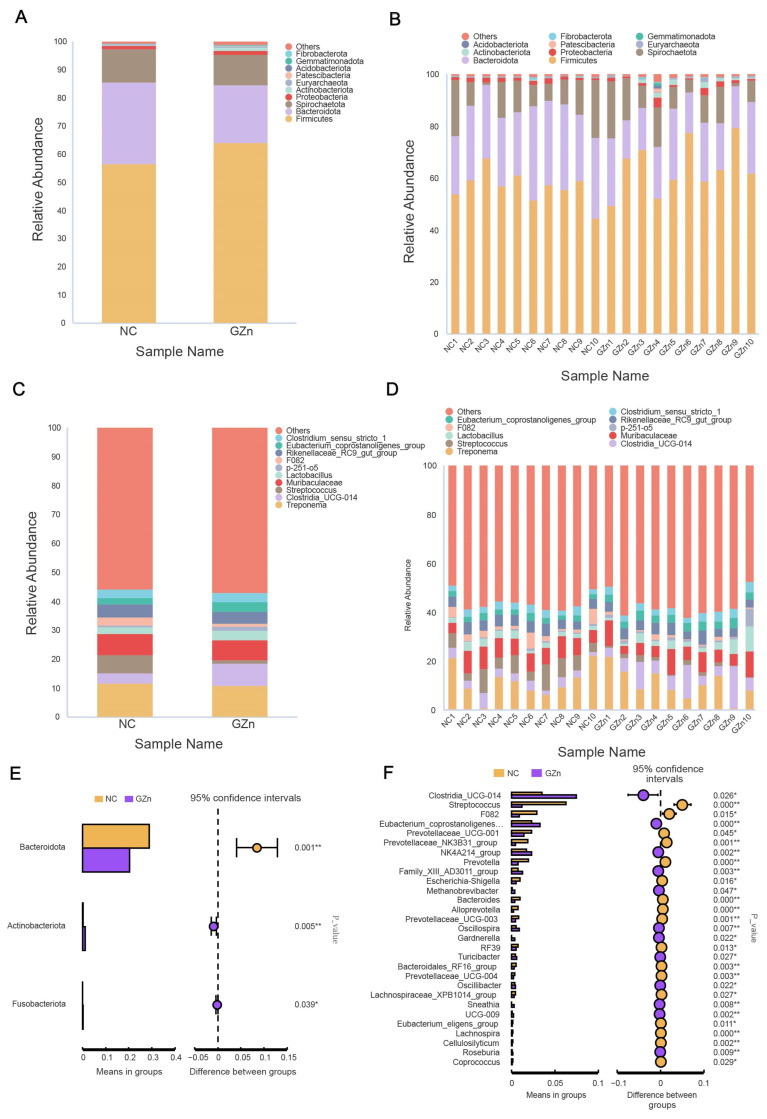
The taxonomic distribution between NC group and GZn group samples (each color represents the relative abundance of a taxonomic bacterium). (**A**) Between-group at phylum level (top 10). (**B**) at phylum level (top 10). (**C**) Between-group at genus level (top 10). (**D**) at genus level (top 10). (**E**) T-test bar plot of significantly different species between groups at phylum level. (**F**) T-test bar Plot of significantly differed species between groups at genus level. * indicates a significant difference (*p* < 0.05), and ** indicates a highly significant difference (*p* < 0.01).

**Figure 4:**
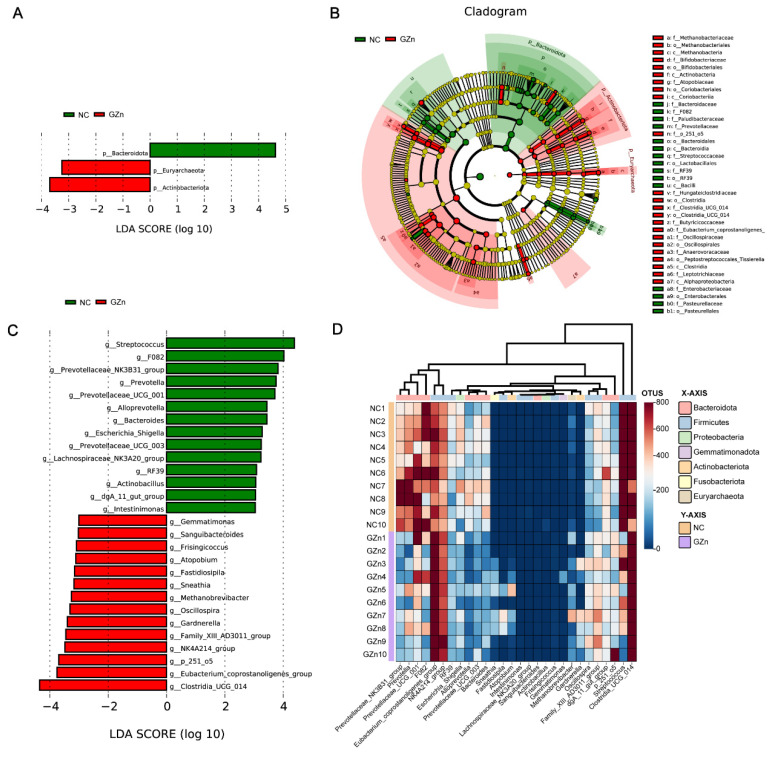
Significant differences in bacterial taxa between NC and GZn groups were determined using linear discriminant analysis (LDA score = 3) and effect size measures (LEfSe). (**A**) Phylum level. NC, nature control. GZn, feeding zinc gluconate. (**B**) LDA tree. (**C**) Genus level. (**D**) Clustering heatmap, showing the OTU content of the top 28 bacterial species with differences at the genus level among samples (LDA > 3).

At the phylum level, a total of 30 phyla were identified across all samples in the NC and GZn groups. Firmicutes was the most abundant phylum in all samples, followed by Bacteroidota. In the NC group, the relative abundances of Firmicutes (56.66 ± 6.11%) and Bacteroidota (28.94 ± 4.29%) reached 85.6%, and Spirochaetota (11.74 ± 6.51%) also had a relatively high abundance. In the GZn group, Firmicutes (64.08 ± 9.92%) and Bacteroidota (20.45 ± 5.03%) remained the most highly abundant, and Spirochaetota (10.91 ± 6.04%) was also relatively high. Interestingly, compared with the NC group, the abundance of Firmicutes in the GZn group significantly increased, while that of Bacteroidota significantly decreased (Figure 3A,B). Multiple T-test analysis revealed that at the phylum level, the relative abundances of Actinobacteriota and Fusobacteriota in the GZn group were significantly higher than those in the control group (*p* < 0.05), whereas the relative abundance of Bacteroidota was extremely significantly lower than that in the control group (*p* < 0.01) (Figure 3E). At the genus level, the core genera in the NC group were *Treponema* (11.72 ± 6.51%), *Muribaculaceae* (7.28 ± 1.63%), *Streptococcus* (6.32 ± 2.61%), *Rikenellaceae_RC9_gut_group* (4.38 ± 0.60%), and *Clostridia_UCG-014* (3.50 ± 1.29%). The genera with higher relative abundances in the GZn group mainly included *Treponema* (10.91 ± 6.04%), *Clostridia_UCG-014* (7.54 ± 4.73%), *Muribaculaceae* (6.84 ± 2.83%), *Rikenellaceae_RC9_gut_group* (4.09 ± 0.87%), and *Eubacterium_coprostanoligenes_group* (3.35 ± 0.53%). Notably, *Clostridia_UCG-014*, which accounted for only 3.50% in the NC group, ranked first among the core genera in the GZn group at 7.54%; meanwhile, *Streptococcus*, which ranked third among the core genera in the NC group, accounted for only 1.24% in the GZn group. This demonstrates that dietary zinc gluconate supplementation significantly altered the microbial proportions at the genus level in the gut microbiota of Yorkshire Pigs (Figure 3C,D). Multiple *t*-test analysis with Benjamini–Hochberg FDR correction showed that the relative abundances of several genera, such as *Clostridia_UCG-014*, were significantly higher in the zinc gluconate group than in the control group (FDR-adjusted *p* < 0.05) (Figure 3F). Based on phylum and genus classifications, LEfSe analysis indicated significant differences in microbial composition between the NC and GZn groups (Figure 4A–C); at the phylum level, compared with the NC group, Euryarchaeota and Actinobacteriota were significantly increased, while Bacteroidota was significantly decreased in the GZn group.

### 3.5. Spearman Analysis

**Figure 5:**
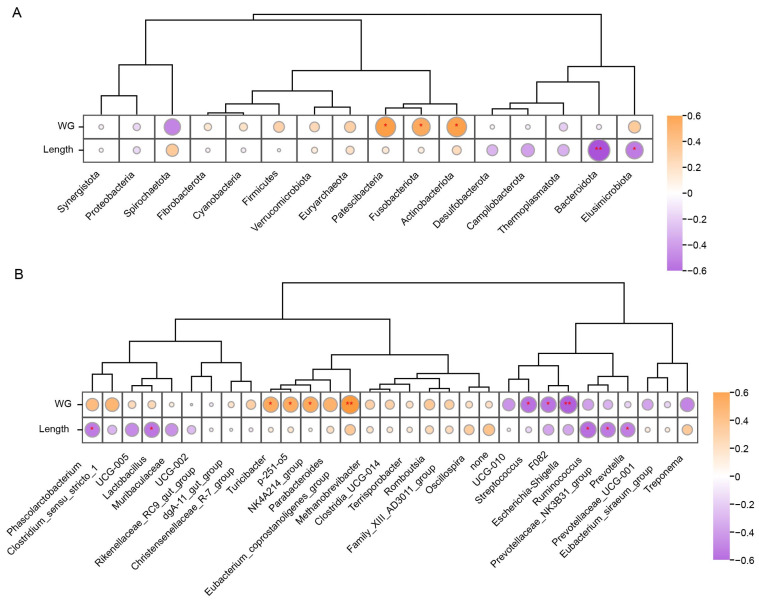
Spearman’s correlation analysis between the gut microbiota and growth performance indicators (WG: weight gain; Length: body length). (**A**) At the phylum level. (**B**) At the genus level. The color of the circles represents the Spearman correlation coefficient (r), with r < 0 indicating a negative correlation and r > 0 indicating a positive correlation. The size of each circle is proportional to the absolute value of the correlation coefficient. * indicates *p* < 0.05, and ** indicates *p* < 0.01.

To explore microorganisms potentially related to growth performance, we conducted Spearman’s rank correlation coefficient analysis between gut microbiota (GM) and weight gain (WG) or body length (Length). At the phylum level, the results showed that Patescibacteria, Actinobacteriota, and Fusobacteriota were significantly positively correlated with weight gain, while Spirochaetota was negatively correlated with it; for body length, Spirochaetota was positively correlated, whereas Elusimicrobiota and Bacteroidota were both significantly negatively correlated (Figure 5A). At the genus level, *Turicibacter* and others were significantly positively correlated with weight gain, whereas *Streptococcus*, *F082*, and *Escherichia-Shigella* were significantly negatively correlated with weight gain (Figure 5B).

### 3.6. Microbial Function Prediction

**Figure 6:**
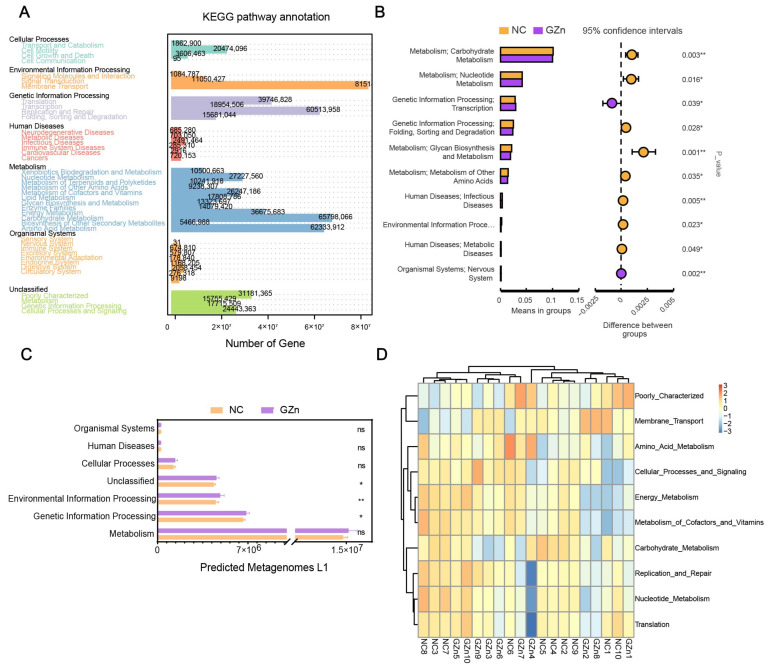
Genome function prediction. (**A**) KEGG pathway annotation. (**B**) T-test. Comparison of NC group and GZn group in different metabolic pathways. (**C**) Histogram of functional predictions based on different groups in the level 1 pathway. Mark * indicates significance test *p* < 0.05, mark ** indicates significance test *p* < 0.01, mark ns indicates significance test *p* > 0.05. (**D**) Heatmap clustering based on the functional predictions for each sample in the 2-level KEGG pathway.

In this study, PICRUSt was used to predict the molecular functions of the microbial communities in both groups of samples. At KEGG Level 2, 41 functional genes were detected in both the NC and GZn groups (Figure 6A). The analysis showed that the gut microorganisms in the NC group were mainly concentrated in pathways such as the Digestive System and Glycan Biosynthesis and Metabolism, whereas those in the GZn group were mainly concentrated in Amino Acid Metabolism, Energy Metabolism, Membrane Transport, and Nervous System pathways (Figure 6C). Through T-tests, we observed significant differences in many KEGG pathways of the fecal microbiota between the two groups (*p* < 0.05). The relative abundances of genes involved in Transcription and the Nervous System were significantly higher in the GZn group than in the NC group (*p* < 0.05), while the relative abundances of genes involved in Carbohydrate Metabolism and Glycan Biosynthesis and Metabolism were significantly lower than those in the NC group (*p* < 0.05) (Figure 6B,D).

## 4. Discussion

The gut microbiota is not merely a microbiome residing within the intestinal tract of humans and animals; it is also referred to as the host’s “second brain.” Previous studies have demonstrated that changes in host animal body weight are closely associated with alterations in their gut microbiota [24]. Identifying gut bacteria that contribute to increased body weight and length is of great significance for promoting healthy animal growth and efficient production [25]. The present study indicates that dietary supplementation with zinc gluconate can significantly increase the body weight and length of Yorkshire Pigs [26]. We utilized 16S rRNA sequencing technology to analyze differences in gut microbial composition and microecological communities among Yorkshire Pigs in different feeding groups. Phenotypic indicators revealed that the pre-slaughter live weight, carcass weight, weight gain, and head weight in the GZn group were all significantly higher than those in the NC group, and body length also showed a significant increasing trend. Based on the results of the Simpson index, species diversity in the GZn group was significantly higher than in the NC group. Additionally, the Chao1 index showed an increasing trend, suggesting that zinc gluconate intervention may have the potential to support gut microecological richness, although this change did not reach statistical significance [27,28].

At the phylum level, Firmicutes and Bacteroidota remained the dominant phyla in both groups. The relative abundance of Bacteroidota in the GZn group was significantly lower than that in the NC group. While our correlation analysis revealed a negative association between Bacteroidota and body length, and previous studies have associated shifts in these phyla with energy metabolism and nutrient utilization in pigs [3,5], the biological significance of these compositional shifts remains context-dependent. Therefore, the hypothesis that zinc gluconate promotes body length by down-regulating intestinal Bacteroidota should be interpreted with caution, as it relies on correlative relative abundance data [29]. Conversely, Actinobacteriota and Fusobacteriota were both significantly positively correlated with Yorkshire Pig weight gain, and the relative abundances of these two phyla in the GZn group were significantly higher than those in the control group. This may be one of the macroscopic taxonomic factors contributing to the significant increase in weight gain and body length in the GZn group.

At the genus level, *Clostridia_UCG-014* emerged as the most highly abundant genus in the GZn group, increasing by 4.04% compared to the NC group. Spearman correlation analysis showed that this genus was significantly positively correlated with both weight gain and body length in Yorkshire Pigs. Meanwhile, the relative abundance of *Streptococcus* was significantly negatively correlated with Yorkshire Pig weight gain and body length, and dietary supplementation with zinc gluconate effectively inhibited the enrichment of this genus [23]. Furthermore, we observed a significant reduction in the relative abundance of Escherichia-Shigella, a genus known to contain opportunistic swine pathogens. The enrichment of taxa such as *Clostridia_UCG-014* and the reduction in genera including Streptococcus and *Escherichia-Shigella* may indicate a modulation of microbial populations potentially related to host performance. However, these associations are based on correlation analyses and do not imply causality. While these compositional shifts are statistically associated with improved growth performance, it must be noted that our 16S rRNA sequencing data only reflects relative compositional changes at the genus level, which cannot account for variations in the total microbial load [30]. Because all experimental pigs remained clinically healthy without signs of diarrhea throughout the study, and we did not evaluate direct health indicators—such as absolute pathogen loads, intestinal barrier integrity, or systemic inflammatory cytokines [31]—these correlations should be interpreted with caution. We cannot definitively conclude whether the suppression of potential pathogens or the enrichment of potential probiotics directly alters growth, or if it is a secondary reflection of an altered gut microenvironment. Further studies integrating microbiome data with functional and physiological measurements, such as absolute quantification (e.g., qPCR) and immunological parameters, are required to confirm these relationships [32].

Notably, the aforementioned beneficial succession at the taxonomic level, particularly the enrichment of core probiotics represented by *Clostridia_UCG-014*, was ultimately validated mechanistically by underlying microecological metabolic functions. PICRUSt functional prediction analysis revealed that the gut microbiota of the GZn group exhibited significant enrichment in pathways such as amino acid metabolism and energy metabolism [33]. Combined with previous studies, the robust activity of these two metabolic pathways is precisely the key driver of host energy harvest [34]. As important members of the Firmicutes phylum, taxa such as *Clostridia* and *Eubacterium* have been reported to possess active amino acid fermentation capabilities [35]. Previous studies indicate that these bacteria can utilize unabsorbed amino acids to produce short-chain fatty acids (SCFAs) [26,27], which may subsequently enter the tricarboxylic acid (TCA) cycle to provide an additional energy source for the host [36,37]. However, it is important to emphasize that our functional data are derived from 16S rRNA gene-based predictions (PICRUSt), and we did not directly measure fecal or serum SCFA concentrations. Therefore, the enhanced SCFA production and subsequent energy harvest discussed here represent a hypothesis rather than a confirmed mechanism. Future studies employing targeted metabolomics (e.g., GC-MS) are necessary to quantify actual SCFA levels and definitively validate whether zinc gluconate supplementation alters these specific metabolic outputs [33,38].

In summary, although the differences in Yorkshire Pig growth phenotypes are influenced by multiple intertwined factors, the shifts in gut microecological composition and predicted metabolic functions observed in this study present a strong statistical association with the enhanced growth phenotypes. While we propose a potential framework linking microecological succession to enhanced metabolic potential, we acknowledge that these 16S-based correlative findings cannot definitively confirm biological mechanisms without direct physiological validation.

## 5. Conclusions

This study comparatively analyzed the fecal microbiota of Yorkshire Pigs in the control group (NC) and the zinc gluconate treatment group (GZn). The results demonstrate that significant differences exist in body weight and body length phenotypes between the two groups of Yorkshire Pigs. Their gut microbial composition also underwent significant succession, and multiple core microorganisms closely related to growth traits were successfully identified. This study provides new insights into the microecological associations accompanying dietary zinc gluconate supplementation in Yorkshire Pigs, but also provides a crucial basis for a deeper understanding of the interactions among “diet-microbiota-host”. More importantly, this study successfully identified key biomarker microbiota associated with improved growth, laying a theoretical foundation and providing broad application prospects for exploring specific organic zinc for nutritional microecological regulation in the future, thereby potentially reducing antibiotic dependence and improving comprehensive economic benefits in animal husbandry.

## Figures and Tables

**Table 1:** Main body size parameters of the Yorkshire Pigs used in this study.

Index	NC	GZn	*p*-Value
Weight before feeding, kg	70 ± 3.44	71.61 ± 4.35	0.4906
Live weight, kg	101.83 ± 8.55	113.22 ± 5.47	0.0118
Torso weight, kg	76.92 ± 6.64	83.94 ± 3.98	0.0331
Weight gain, kg	31.83 ± 9.33	41.61 ± 5.08	0.0300
Length, cm	114.33 ± 3.73	118.89 ± 4.25	0.0685
Heart, kg	0.43 ± 0.06	0.48 ± 0.06	0.1707
Liver, kg	1.50 ± 0.17	1.57 ± 0.23	0.5237
Spleen, kg	0.18 ± 0.03	0.18 ± 0.02	0.8734
Head, kg	5.99 ± 0.36	6.47 ± 0.29	0.0205
Hind hoof, kg	0.56 ± 0.04	0.59 ± 0.04	0.2323

Note: The data are expressed as mean ± standard deviation.

**Table 2:** The alpha diversity index of each sample.

Sample	Chao1	Shannon	Simpson	Good’s Coverage
NC1	777.82	7.44	0.974	0.99938
NC2	831.13	8.01	0.990	0.99941
NC3	855.63	7.98	0.987	0.99965
NC4	843.44	8.07	0.989	0.99952
NC5	826.89	7.70	0.985	0.99959
NC6	910.43	8.08	0.990	0.99924
NC7	807.93	8.04	0.987	0.99959
NC8	828.69	7.81	0.984	0.99945
NC9	812.97	8.00	0.990	0.99959
NC10	734.48	7.24	0.971	0.99972
GZn1	796.93	7.21	0.958	0.99955
GZn2	772.11	7.05	0.958	0.99952
GZn3	815.57	7.45	0.976	0.99931
GZn4	1230.49	8.05	0.978	0.99903
GZn5	928.25	8.21	0.990	0.99948
GZn6	845.65	6.95	0.945	0.99910
GZn7	932.17	8.16	0.991	0.99917
GZn8	850.24	7.48	0.973	0.99931
GZn9	941.32	7.49	0.967	0.99938
GZn10	923.12	7.89	0.987	0.99945

## Data Availability

The data supporting the findings of this study are included within the article. Further inquiries can be directed to the corresponding author.

## Abbreviations

The following abbreviations are used in this manuscript:

GM	Gut Microbiota
GZn	Zinc gluconate
OTU	operational taxonomic unit
PCoA	principal coordinates analysis
LDA	Linear discriminant analysis
NMDS	the Non-metric Multidimensional Scaling
WG	weight gain
SCFAs	short-chain fatty acids
TCA	tricarboxylic acid
